# Improving Consensus Scoring of Crowdsourced Data Using the Rasch Model: Development and Refinement of a Diagnostic Instrument

**DOI:** 10.2196/jmir.7984

**Published:** 2017-06-20

**Authors:** Christopher John Brady, Lucy Iluka Mudie, Xueyang Wang, Eliseo Guallar, David Steven Friedman

**Affiliations:** ^1^ Dana Center for Preventive Ophthalmology Wilmer Eye Institute Johns Hopkins University School of Medicine Baltimore, MD United States; ^2^ Bloomberg School of Public Health Department of Epidemiology Johns Hopkins University Baltimore, MD United States

**Keywords:** crowdsourcing, diabetic retinopathy, Rasch analysis, Amazon Mechanical Turk

## Abstract

**Background:**

Diabetic retinopathy (DR) is a leading cause of vision loss in working age individuals worldwide. While screening is effective and cost effective, it remains underutilized, and novel methods are needed to increase detection of DR. This clinical validation study compared diagnostic gradings of retinal fundus photographs provided by volunteers on the Amazon Mechanical Turk (AMT) crowdsourcing marketplace with expert-provided gold-standard grading and explored whether determination of the consensus of crowdsourced classifications could be improved beyond a simple majority vote (MV) using regression methods.

**Objective:**

The aim of our study was to determine whether regression methods could be used to improve the consensus grading of data collected by crowdsourcing.

**Methods:**

A total of 1200 retinal images of individuals with diabetes mellitus from the Messidor public dataset were posted to AMT. Eligible crowdsourcing workers had at least 500 previously approved tasks with an approval rating of 99% across their prior submitted work. A total of 10 workers were recruited to classify each image as normal or abnormal. If half or more workers judged the image to be abnormal, the MV consensus grade was recorded as abnormal. Rasch analysis was then used to calculate worker ability scores in a random 50% training set, which were then used as weights in a regression model in the remaining 50% test set to determine if a more accurate consensus could be devised. Outcomes of interest were the percent correctly classified images, sensitivity, specificity, and area under the receiver operating characteristic (AUROC) for the consensus grade as compared with the expert grading provided with the dataset.

**Results:**

Using MV grading, the consensus was correct in 75.5% of images (906/1200), with 75.5% sensitivity, 75.5% specificity, and an AUROC of 0.75 (95% CI 0.73-0.78). A logistic regression model using Rasch-weighted individual scores generated an AUROC of 0.91 (95% CI 0.88-0.93) compared with 0.89 (95% CI 0.86-92) for a model using unweighted scores (chi-square *P* value<.001). Setting a diagnostic cut-point to optimize sensitivity at 90%, 77.5% (465/600) were graded correctly, with 90.3% sensitivity, 68.5% specificity, and an AUROC of 0.79 (95% CI 0.76-0.83).

**Conclusions:**

Crowdsourced interpretations of retinal images provide rapid and accurate results as compared with a gold-standard grading. Creating a logistic regression model using Rasch analysis to weight crowdsourced classifications by worker ability improves accuracy of aggregated grades as compared with simple majority vote.

## Introduction

### Overview

Diabetes mellitus (DM) is a highly prevalent disease affecting over 415 million individuals worldwide, 80% of whom reside in low- and middle-income countries [1]. By 2040, the prevalence of DM is expected to reach 642 million, with the largest increases seen in countries with developing economies [1]. In the United States, 21.0 million people had known diabetes in 2012 and another 8.1 million had undiagnosed diabetes [2]. Diabetic retinopathy (DR) is an important complication of DM, currently affecting approximately 93 million people worldwide, with 28 million of these suffering from vision-threatening DR [3]. It is estimated that the number of Americans with DR will reach 16 million by 2050, with 3.4 million of these individuals afflicted with vision-threatening DR [4].

While DR is the leading cause of vision loss in working age individuals [4], screening for DR is an effective and cost-effective means of identifying the disease early, referring affected individuals for appropriate therapies, and preventing vision loss [5-8]. Despite the increasing prevalence of DR, the annual increase in the number of practicing ophthalmologists is only 2% [9], largely in high-income countries [10]. As a way of overcoming human resource shortfalls and in order to increase adherence with DR screening recommendations more broadly, telehealth programs using nonmydriatic fundus photography and remote interpretation are increasing [11-13].

In addition to improving screening uptake, telehealth may provide ways to reduce provider, payer, and societal costs [14-16]. Among the costs of a telehealth program for DR screening are the fundus camera, the telehealth software package, and the human resources needed for image acquisition and interpretation. Fundus photo interpretation costs in DR screening may be high given labor-intensive interpretation protocols and the need to interpret multiple images per patient. Computerized, semiautomated image analysis techniques have been developed which may be able to reduce physician workload and screening costs [17-19]; however, these methods are not approved by the US Food and Drug Administration nor in wide use clinically at this time. As telehealth expansion continues, novel low-cost methods will be needed to interpret the large volume of fundus images expected with rising incidence of diabetes, especially in resource-poor settings and in large public health screenings.

The use of crowdsourcing in biomedical research is in its infancy, although some groups have used this method in public health research [20] and to interpret biomedical images [21,22]. Crowdsourcing has been used to categorize a number of fundus photos with a variety of diagnoses as normal or abnormal [23]. In a trial conducted in the United Kingdom using untrained graders, the sensitivity was ≥96% for normal versus severely abnormal and from 61% to 79% for normal versus mildly abnormal [23]. In a proof-of-concept study, we have demonstrated that untrained crowdsourced workers can rapidly and accurately identify images with DR [24]. We have also demonstrated that crowdsourcing workers can improve their ability to identify characteristic glaucomatous changes in optic nerve photographs [25]. In this study we seek to perform an external validation of our method of crowdsourcing DR identification using a public dataset of 1200 retinal photographs and explore methods of improving the determination of a consensus score from multiple individual crowdsourced grades including creating a logistic regression model that includes other data points collected at the time of the grading and a second model that weights the responses of graders based on ability in a training dataset using the Rasch model.

### Crowdsourcing Background

Crowdsourcing is “an online, distributed problem-solving and production model that leverages the collective intelligence of online communities to serve specific organizational goals” [26]. Distributed human intelligence tasking [26], a subset of crowdsourcing, can involve subdividing larger tasks into small portions and then recruiting a group of individuals to each complete these small portions, and only collectively, the entire task. Amazon Mechanical Turk (AMT) is an online distributed human intelligence market that allows access to thousands of people who can quickly accomplish small, discrete tasks for small amounts of money. Typical AMT tasks include tagging photos, translating words, or writing very short articles for websites. AMT has its own vocabulary used by workers (Turkers) and task administrators (Requestors). A human intelligence task (HIT) is a small job which may be performed in a matter of seconds or minutes and, once the work is approved by the Requestor, may pay $0.01 to $0.25 or more per task depending on the complexity of the HIT. A group of HITs is called a batch and is made up of similar HITs. Depending on the complexity of the task and the payment offered by the Requestor, a batch is often completed within minutes or hours of posting [27]. One particular application in the recent literature has been the use of crowdsourcing to generate ground-truth annotations for deep learning algorithm training and validation [28], which have themselves been recently demonstrated to be quite effective at DR retinal image classification [28,29]. Other types of crowdsourcing such as broadcast search have also been applied to retinal image grading through a 2015 Kaggle competition [30].

### Finding the Consensus Grade

Several methods for aggregating multiple grades into a consensus score have been described in the biomedical literature, dating back several decades [31]. The simplest method, termed majority vote (MV), involves promoting the modal response to the crowdsourced determination, as described in Whitehill et al [32]. In a binary classification scheme, whichever response is selected by half or more of respondents becomes the consensus. While this approach is computationally simple, the differential ability of workers is ignored as is differential difficulty of the unique tasks. Therefore, other methods of aggregating scores have been explored that rely on patterns of individual worker responses over multiple tasks and comparisons with or incorporation with expert annotations where available [22,32-36]. Additionally, several investigators have explored incorporation of artificial intelligence or deep learning methodologies to aggregation of data [37]. Others have turned to methods of item response theory to improve aggregation by specifically looking for inattentive or malicious users and eliminating their data [38]. In this study, we hypothesized an improved consensus grade would be found using Rasch analysis–determined weights applied to logistic regression models.

## Methods

### Crowdsourcing Platform

An interface for fundus photo classification has been previously described for the AMT crowdsourcing platform [24]. The United Kingdom national screening program grading scale [39] was chosen due its broad clinical telemedicine deployment. For the purposes of the study, terms from this scale were translated into plain language: background retinopathy was called mild, preproliferative retinopathy was called moderate, and proliferative retinopathy was called severe. Maculopathy is defined as abnormal on a training image with otherwise moderate disease but is not coded separately. The AMT interface was designed to provide training on grading of DR within each HIT. This training includes 7 images annotated with the salient features of each level of retinopathy in plain language. Turkers are presented with the following text: “This is a photo of the inside of the eye. We are looking to label eyes as healthy or unhealthy with respect to diabetes. Rate this eye.” Turkers can hover their mouse over the adjacent training images (2 normal, 1 mild, 1 moderate, 3 severe) while reviewing the active test image (Multimedia Appendix 1). This layout allows for all of the training and grading to occur in one browser window. Turkers receive US $0.10 per image, with a 40% commission going to Amazon, for a total cost of US $1.40 per image.

### Baseline Trial with Majority Vote Analysis

For the first phase of this project, 1200 images from the Messidor public dataset [40] were posted for 10 unique binary annotations to provide external validation of the prior proof-of-concept study (yielding a dataset of n=12,000). We previously found 10 annotations per image appears to produce the maximal area under the receiver operating characteristic (AUROC), with little benefit seen for >10 gradings per image [24]. The Messidor dataset is composed of 800 mydriatic and 400 nonmydriatic retinal fundus photos of universally high quality and resolution. The images are supplied with ground truth grading on the following scale:

- 0—normal: no microaneurysms, no hemorrhages (n=546)
- 1—1-5 microaneurysms, but no hemorrhages (n=153)
- 2—6-14 microaneurysms OR 1-4 hemorrhages, but no neovascularization (n=247)
- 3—15 or more microaneurysms OR 5 or more hemorrhages OR presence of neovascularization (n=254)

For the purposes of this study, the presence of 5 or fewer microaneurysms was felt to be clinically insignificant and thus we classified Messidor 0 and 1 images as normal (58%, n=699) and Messidor 2 and 3 images as abnormal (42%, n=501), to mimic the accuracy of an American Telemedicine Association Category 1 screening program [41]. Turkers were not made aware of the source of the images.

To create the MV consensus, each image was assigned the grade of abnormal if half or more Turkers deemed an image abnormal, otherwise the image was classified as normal. Sensitivity, specificity, and AUROC were calculated. This batch and grading scheme served as the baseline results for comparison with the regression models used in later phases of the research.

As there was no a priori rationale to suggest that the mean Turker score (with rounding toward abnormal) would provide the most accurate approximation of the ground truth classification, additional methods of generating consensus were explored.

### Weighted Logistic Regression Model

**Figure 1:**
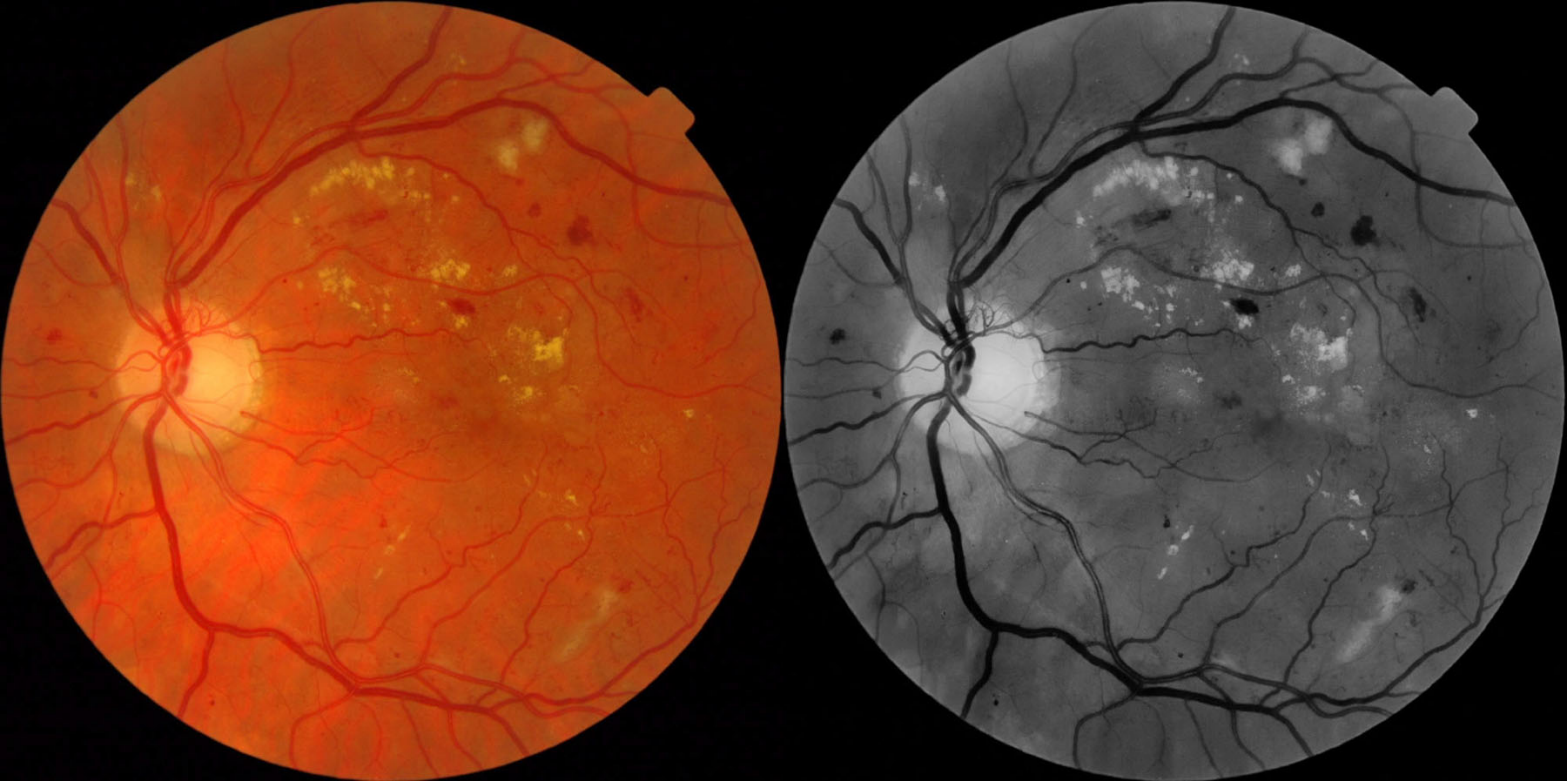
Example color image and simulated red-free retinal photograph created by deleting the red channel in Adobe Lightroom.

For this phase, we recognized that among Turkers there is a range of ability, and among images there is a range of difficulty. In order to improve throughput, Turkers were asked to grade images in multiples of 10 (rather than single images), and to collect more data about the Turkers’ interactions with the task for future phases, the project was migrated to a new online interface using Volunteer Science (Multimedia Appendix 2). In the new interface, the 1200 Messidor images were posted for binary grading first using the full color images and then again with the images converted to grayscale with the red color channel removed in Adobe Lightroom (applied B&W Preset with green filter, in B&W Mix reduce red to –75) (Figure 1). This was done to simulate red-free images, which may allow for better detection of DR [42]. This allowed us to have a dataset with up to 30 grades per image across the 3 different batches (baseline, phase 2 color, and phase 2 black and white), albeit captured under slightly different circumstances. Throughout, Turkers were paid $0.10 per image.

**Figure 2:**
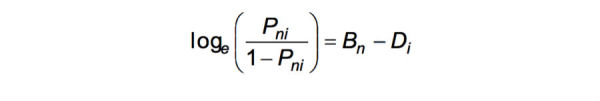
The Rasch model formula.

The dataset of 1200 images was randomly divided into 600 training and 600 test images. The distribution of Messidor categories within these 2 subsets was within 2% of the entire dataset. Using the training images, a matrix of images and individual Turkers was created with each cell either being a missing datapoint (if that particular Turker did not grade that particular image), a 1 for a correct classification, or a 0 for an incorrect classification. Rasch analysis was then performed to determine the image measures and Turker measures based solely on the information in this matrix in the training set (see Figure 2 for the Rasch model [43], where, in this study, *P*_ni_ is the probability of a given image *n* of difficulty *B*_n_ having a correct response provided by Turker *i* of skill level *D*_i_). Therefore, the Turker’s ability measure and the image’s difficulty measure are expressed as log-odds units (logits), theoretically ranging from –∞ to +∞. The negative exponentiated Turker ability measure, then, is the odds that an image of average difficulty (ie, *B*_n_=0) would be categorized correctly by that particular Turker. This value was then multiplied by each of that Turker’s categorizations from the test set (with abnormal =1, normal =–1). The weighted scores were then summed for each image. In an initial analysis, the consensus image score was considered to be abnormal if greater than or equal to zero and normal otherwise. Sensitivity, specificity, and AUROC were calculated as above with comparison to the baseline MV results. In a subsequent analysis, the consensus image score was included as a continuous variable in a logistic regression model to determine the ideal cut-off value for different values of percent correct, sensitivity, and specificity.

Data were analyzed using Stata Statistical Software: Release 14 (StataCorp LLC) and Winsteps Rasch measurement computer program (winsteps.com). The Johns Hopkins University Institutional Review Board (IRB) deemed this research IRB-exempt as nonhuman subjects research.

## Results

### Baseline Majority Vote

A batch of 12,000 (1200 images × 10 repetitions) tasks was posted on AMT March 13, 2015, 11:00 AM Eastern Time for a total cost of US $1440 ($1200 for Turker compensation, $240 for Amazon commission). The grading was complete in 68 minutes, with 97% of tasks (11,640/12,000) graded within 35 minutes. Tasks submitted without image grades were immediately reposted so there were no missing data. The tasks were submitted by 281 unique Turkers, with each submitting a mean of 42.7 tasks (median 28, mode 1). Turkers were only able to grade each image once.

**Figure 3:**
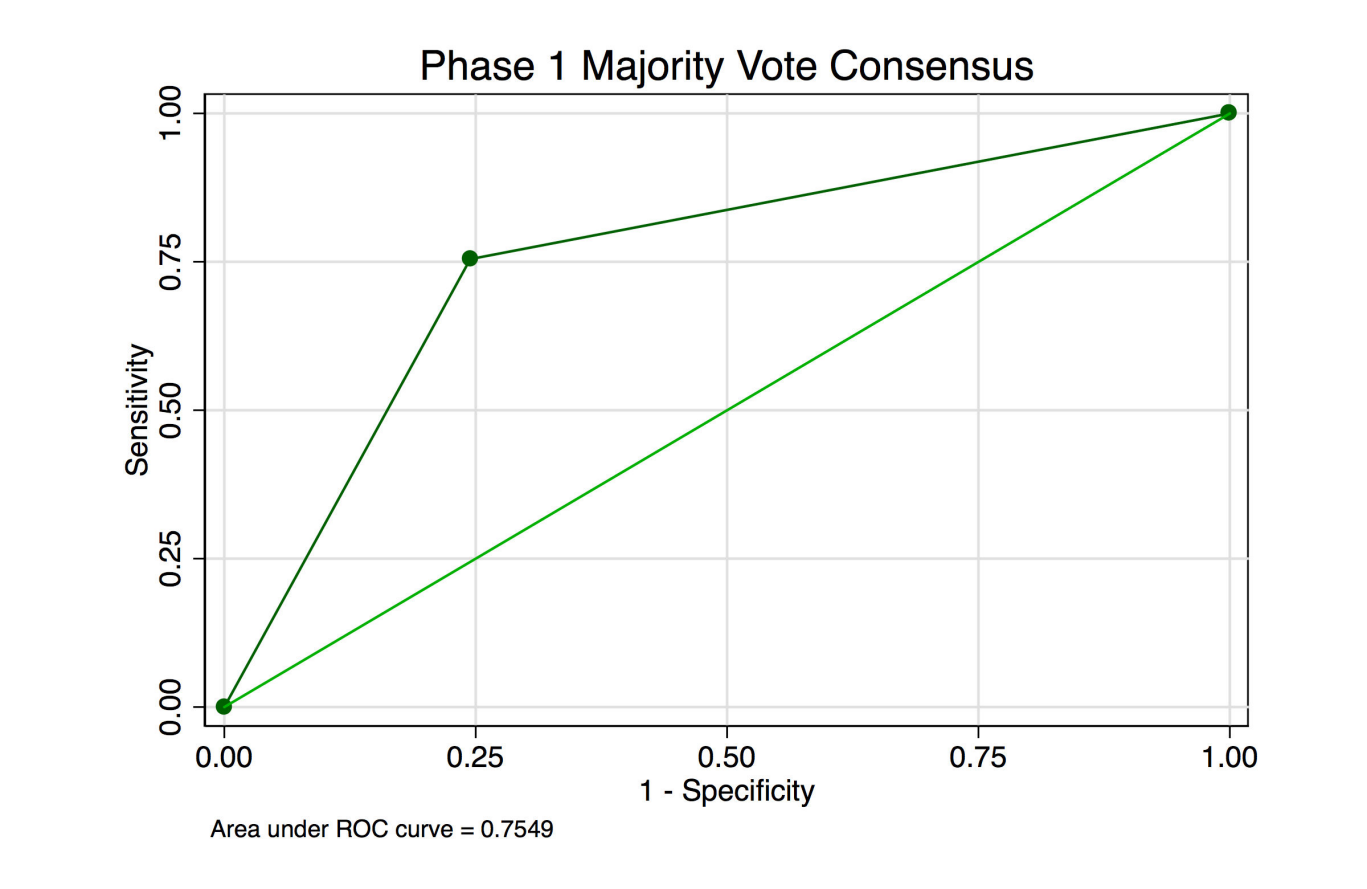
Receiver operating characteristic for the diagnosis of abnormal retinal photograph in the phase 1 baseline analysis.

The MV consensus was correct in 75.5% (906/1200) of images. Sensitivity and specificity were both 75.5%. The AUROC was 0.755 (Figure 3).

### Weighted Logistic Regression Model

For this phase, the focus shifted to the perspective of the Turkers rather than on the images themselves. Exploration of Turker accuracy motivated an attempt to incorporate Turker ability into a predictive model. There is expected to be a distribution of Turker accuracy that is not necessarily related to the number of tasks performed [44]. As such, any method that implicitly weights a consensus score based on number of tasks performed as does MV may reduce accuracy. In the phase 1 baseline task, among the 281 unique Turkers median percentage of images graded correctly was 64.7% (18 correct out of the median graded of 28) with an interquartile range of 55.5% to 74.4%.

**Figure 4:**
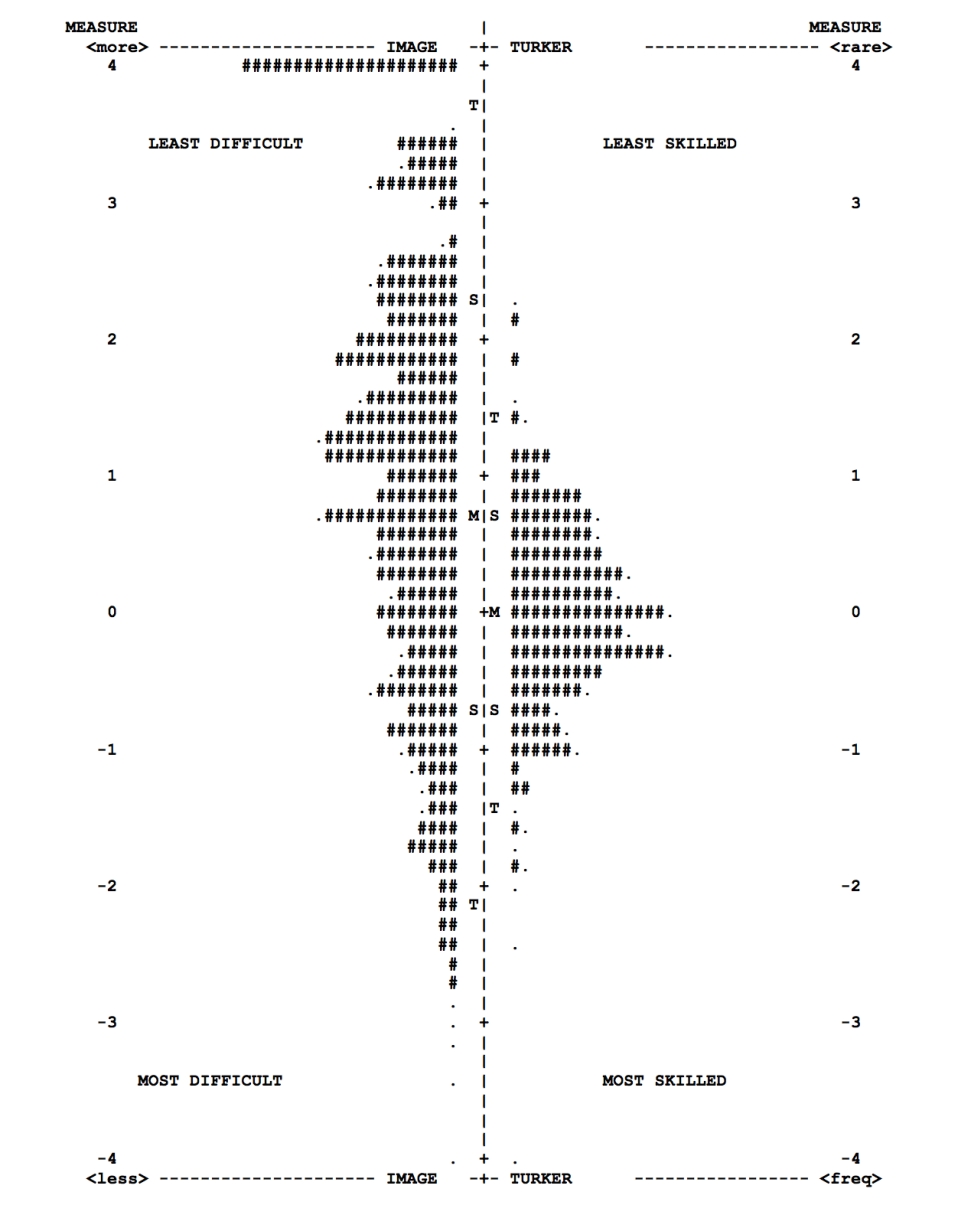
Image-Turker map illustrating distribution of measure scores for image grading difficulty and Turker ability (#=2 images/Turkers, .=1 image/Turker, M=mean score, S=1 standard deviation, T=2 standard deviations).

Prior to performing Rasch analysis, the results of the improved Volunteer Science interface (1200 color images + 1200 red-free images; cost US $2558, completed over 10 days) were merged with the phase 1 baseline classifications to permit as many grades as possible. In essence, we treated the Turkers as test takers taking a test involving grading multiple images. Duplicate grades of the same image by an individual Turker were deleted (6.7%; 2228/33,319 grades deleted). For stability, we also excluded 1027 grades by 227 Turkers who had graded fewer than 10 images within the training set of 600 images (leaving 301/528 Turkers and 14,539/15,566 grades). No images were excluded. Using Rasch analysis, we found Turker ability ranging from the most highly skilled at –3.75 logits to the least skilled at 1.9 logits. The median ability is set in the model as zero, and the interquartile range of ability was –0.40 to 0.47 (Figure 4).

After transformation, the Turker measure scores from log-odds to odds of correctly classifying an average difficulty image, weights outside the top and bottom centiles (1%) were truncated to the level of the 1st and 99th centile to increase stability and minimize the effect of outliers.

When the Turker weights were applied to the classifications in the color images in the test set using an arbitrarily determined cut-off (0), the percent correctly classified improved to 80.7% with an AUROC of 0.817 (from 74.0% correct and AUROC 0.739 for the test set images only in the phase 1 baseline task).

**Figure 5:**
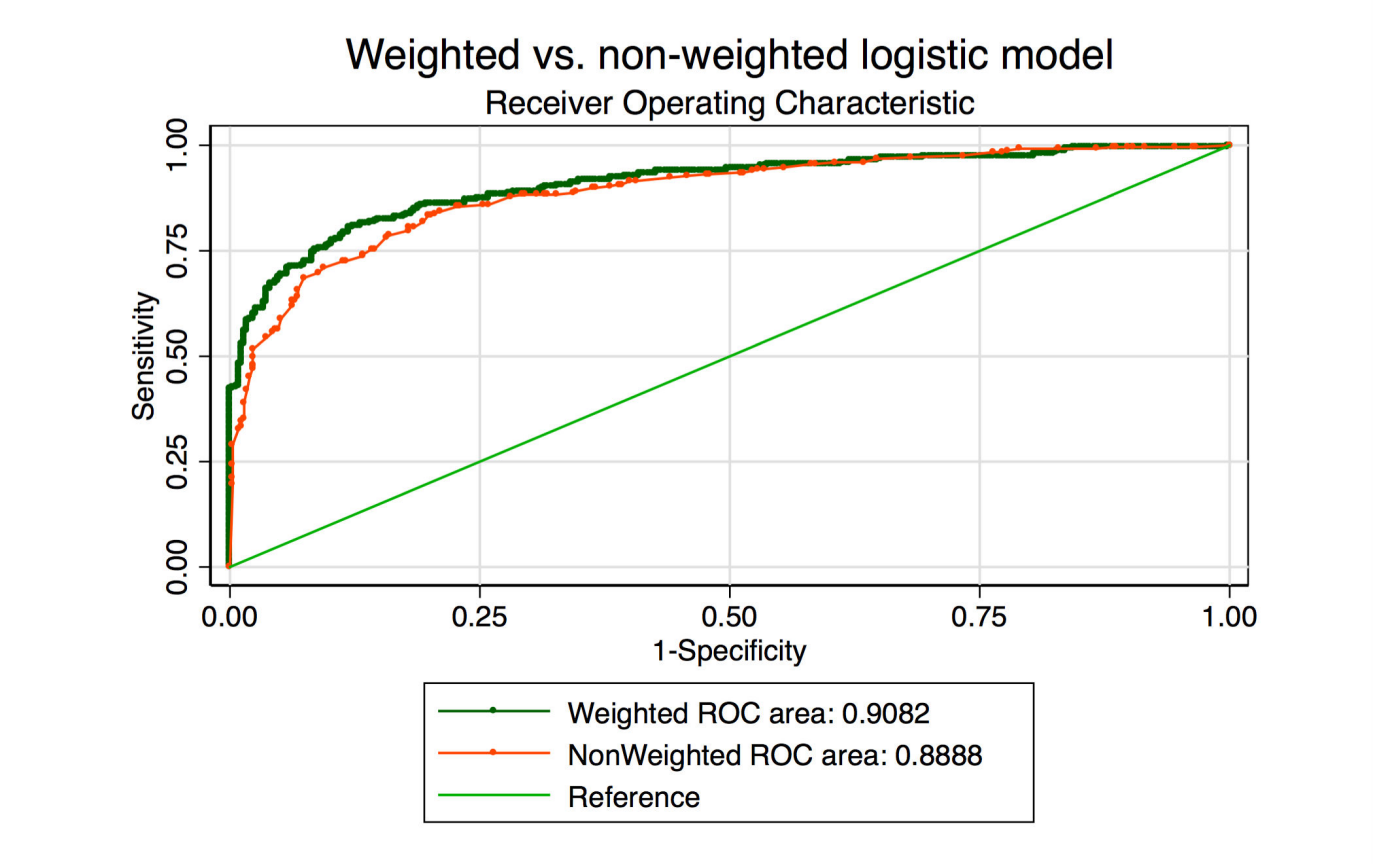
Receiver operating curve generated from a logistic regression model using weighted consensus scores of the random 50% (600 images) test set and a second using the nonweighted scores from the same data.

To determine if the arbitrary cut-off could be improved, a logistic regression model using the consensus image score determined by the weighted Turker classifications was generated. Using this model, a much more granular receiver operating characteristic (ROC) could be generated. A similar ROC was generated from a separate regression model using the unweighted consensus classifications from the same batch (Figure 5). The AUROCs were 0.908 (95% CI 0.883-0.933) and 0.889 (95% CI 0.862-916), respectively (chi-square *P* value<.001). A post hoc sensitivity analysis exploring the decision to dichotomize the Messidor dataset between grades 1 and 2 was performed by excluding all Messidor grade 1 images. In this analysis, the AUROCs were 0.919 (95% CI 0.896-0.943) for the Rasch-weighted scores and 0.888 (95% CI 0.861-0.915) for the unweighted scores (chi-square *P* value<.001). An additional sensitivity analysis was performed by using the entire 1200 images to generate Rasch Turker measures, and then the AUROC was calculated by using a Jackknife cross-validation excluding 1 image each time for 1199 repetitions [45]. Using this method, the AUROC was 0.909 (95% CI 0.892-0.926).

**Figure 6:**
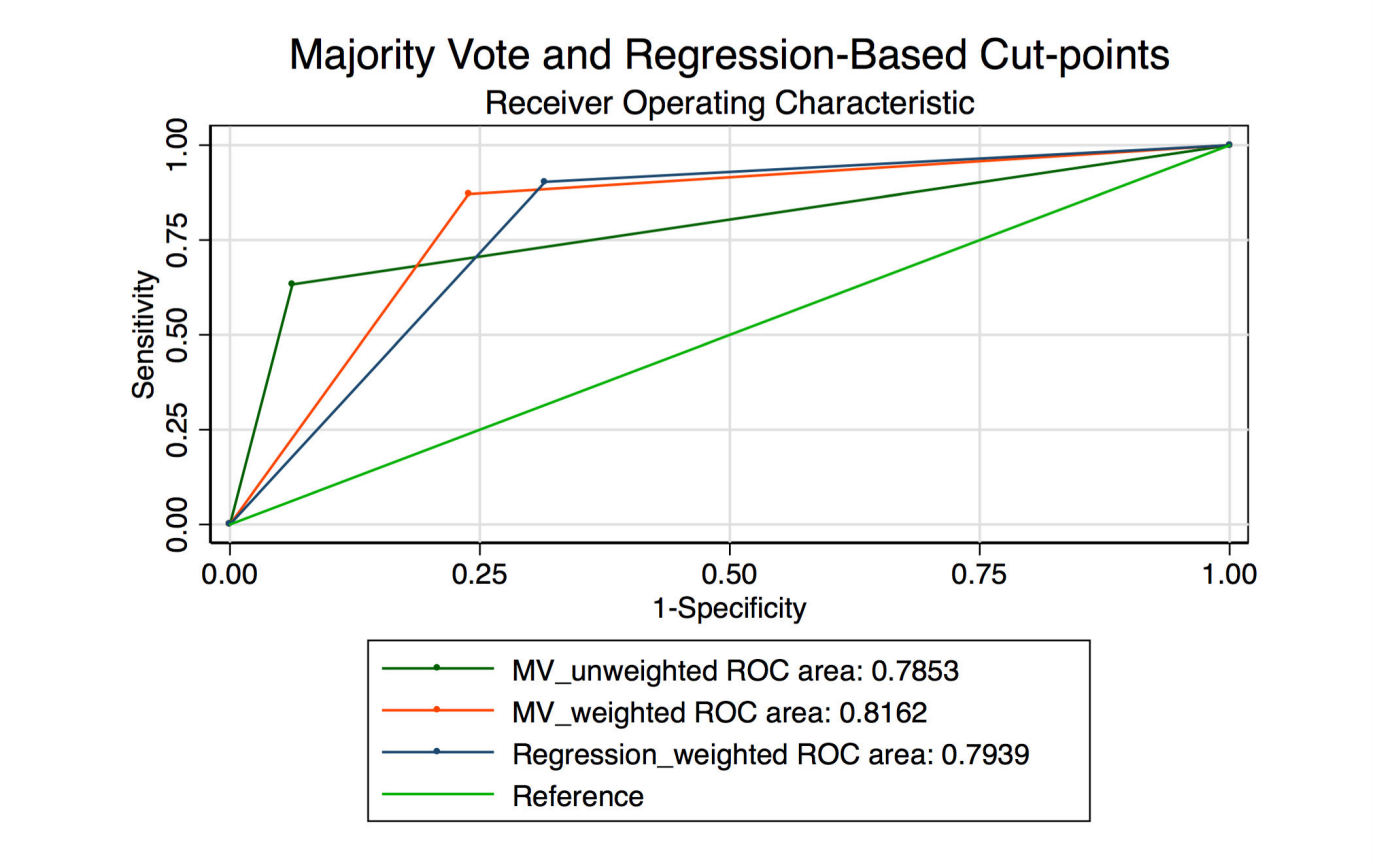
Receiver operating curve from logistic regression model using weighted consensus scores using a dichotomization cut-point designed to permit sensitivity of 90% shown alongside unweighted and Rasch-weighted majority vote cut-points.

Examination of multiple dichotomization cut-points revealed that choosing a cut-off that would permit a minimum sensitivity of 90.3% allows for specificity of 68.5% and percent correctly classified at 77.5% with an AUROC of 0.79 (95% CI 0.76-0.83) (Table 1,Figure 6).

**Figure 7:**
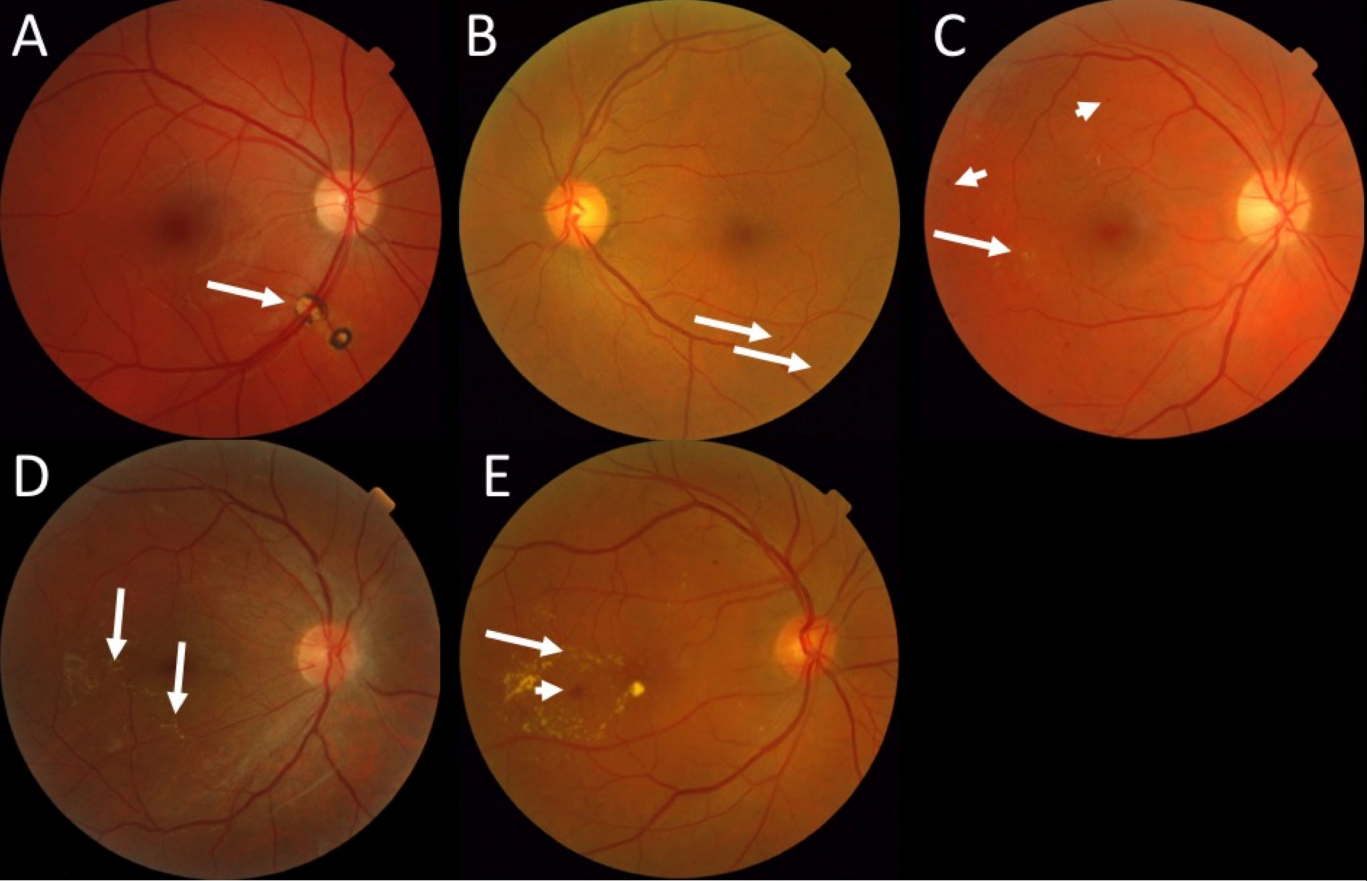
Representative retinal fundus images organized by progressive ease of grading correctly (A-E). (A) The image reveals areas of chorioretinal atrophy (arrow) but is without lesions of diabetic retinopathy. (B) This image reveals very subtle microaneurysms (arrows). (C) This image reveals more obvious microaneurysms (arrowheads) and subtle hard exudates (arrow). (D) This image reveals more apparent hard exudates (arrow). (E) This image reveals obvious hard exudates (arrow) and more obvious hemorrhagic microaneurysms (arrowhead).

Rasch analysis also allowed for a qualitative analysis of the retinal images. The images were sorted by image measure on the logit scale as generated by the Rasch analysis described earlier. The 20 images with the lowest measures ranged from –4.75 to –2.56 logits, which corresponds to the log odds that a Turker of average ability (ie, Turker measure = 0) would grade these images correctly. Among these 20 images, 0, 1, or 2 Turkers (out of maximum of 30) graded each correctly, and thus these images were designated the most difficult to grade. The 20 images with the highest measures were selected as the easiest to grade. A total of 20 sequential images were then selected at the 3 image measure quartiles as successively less difficult images to grade (Table 2,Figure 7). The hardest images were largely Messidor grade 0 and 1 images with some abnormal features but without significant DR (eg, chorioretinal atrophy, choroidal nevus) that had been graded as abnormal by Turkers. Intermediate images were mostly Messidor grade 2 images with extrafoveal microaneurysms of subtle hard exudates as well as Messidor grade 0 images without any nondiabetic pathology or distracting features. The easiest images were generally Messidor grade 3 with prominent hard exudates apparent.

Because data on the time spent completing the task and prior exposure to similar tasks is collected in addition to the grade for the current image task when a crowdsourcing worker completes a task, a separate logistic regression model that incorporated variables for time spent on each task and prior experience with ophthalmic HITs was run but did not improve diagnostic accuracy (data not shown).

**Table 1:** Characteristics of different cut-point values using the weighted logistic model, as compared with the majority vote weighted cut-point and the phase 1 baseline task.

		Correct %	Sensitivity %	Specificity %	AUROC^a^ 95% CI
Phase 1 MV^b^ baseline		75.5	75.5	75.5	0.75 (0.73-0.78)
MV weighted arbitrary cut-point		80.7	87.1	76.1	0.82 (0.79-0.85)
**Weighted regression**					0.91 (0.88-0.93)
	Maximizing % correct	85.0	81.1	87.8	0.84 (0.81-0.87)
	Sensitivity ≈ 90%	77.5	90.3	68.5	0.79 (0.76-0.83)
	Specificity ≈ 90%	84.5	76.6	90.1	0.83 (0.80-0.86)

^a^AUROC: area under the receiver operating characteristic.

^b^MV: majority vote.

**Table:** 

Difficulty	Measure score range logits	Images graded correctly %	Messidor grade mode
Hardest	–4.74 to –2.56	0-8.3	1
Intermediate 1	–0.14 to –.04	43.4-53.9	0
Intermediate 2	1.01-1.1	69.2-76.9	0
Intermediate 3	2.04-2.09	85.2-88.9	0
Easiest	4.5-4.91	100	3

## Discussion

We have shown that workers on a popular crowdsourcing platform AMT are able to rapidly and accurately identify mild to moderate DR in a large public dataset and that weighting Turker responses by their demonstrated ability improves the accuracy of their crowdsourced grades.

There are many ways of defining a crowdsourcing consensus, or “divining the wisdom of the crowd.” For binary tasks or categories that can be rationally dichotomized (as was done in this study by reducing 4 levels of disease to disease or no disease), one could take a simple MV approach such that the image receives the categorization rendered by half or more of respondents. To reach a consensus with categorical data, using the modal response may reduce the influence of outlier or inattentive/malicious users. Both methods involve a post hoc analysis of the data. Alternatively, one could allow consensus to be determined on the fly, such that if enough workers render the same or similar judgment of an image, the image is immediately coded with this classification so that the full 10 responses need not be completed.

In this study, we sought to determine whether knowledge of an individual Turker’s ability on a training set of images could be used to improve accuracy of the consensus grade in a separate test set of images. We chose to use the Rasch model with image difficulty as the latent trait. In this way, we were able to determine the odds of each image being correctly classified by a Turker of average ability and the odds of each Turker being able to correctly grade an image of average difficulty. Using the entire 600 image training set, we saw reasonable targeting of Turker ability and image difficulty. This allowed us to weight a Turker’s response to the images in the test set for use in a logistic regression model. This also allowed for a qualitative assessment of the retinal images from a unique perspective, ranked from difficulty to grade correctly rather than ranked by disease severity.

While we were not able to meet all assumptions of the Rasch model for this study, we noted that the use of weighting Turkers’ responses showed a small but significant improvement in the AUROC as compared with unweighted aggregation. This result was very encouraging and suggests several possible improvements that can be made to our crowdsourcing method. For example, if a returning Turker has previously had their ability calculated, this can be immediately applied to their new categorizations. If a new Turker begins a retinal grading task, they can be asked to perform a brief quiz to determine their ability prior to officially grading images. This method may allow for a reduction in the number of annotations per image required to generate a stable estimate for each image. Moreover, we believe the use of regression methods to be of benefit as they allow diagnostic cut-points to be set based on the specific needs of the clinical or research paradigm (ie, to select the balance between sensitivity and specificity). We have also demonstrated the utility of a brief training prior to completing annotation of glaucomatous optic nerve images [25]. The relative (or combined) utility of these 2 approaches remains to be tested.

Additionally, in a recent study exploring the use of deep learning artificial intelligence for retinal image interpretation published by researchers at Google [28], a stated limitation was their use of MV consensus grading of several ophthalmologists in both their 128,000+ image training set and 11,700+ image test sets. The authors acknowledged that much of the residual imprecision of the algorithm likely resides in feeding better gold standard data into the algorithm, creating an opening for similar methods as described here.

There are several limitations to crowdsourcing retinal image processing. Because users are anonymous and cannot be directly selected by the researcher, there is no way to ensure high quality, conscientious workers each time work is posted. Since we did not collect demographic information from our Turkers, there was no way to use Turker factors to predict worker accuracy. Indeed, the pool of workers can vary substantially over time and different trends in how workers engage with the site have become apparent to us over the course of the 3 years of this experiment. For example, we have recently noticed that many workers use automated scripts to accept or reserve large numbers of tasks at once, and then they can proceed at their own pace without concern for there being few tasks left for them. This hoarding has made metrics of time spent per image rather meaningless, but it is not clear that it has led to worse outcomes overall (data not shown). Regardless, researchers who wish to use crowdsourcing need to be aware of the culture of the crowdsourcing marketplace they choose.

Our current method used the supplied Messidor grade as the gold standard. While this is a high-quality, well-known dataset, there were dramatic differences in how the images were graded compared to standard clinical and telemedicine grading schemes such as the one we used for training. Particularly, while we tried to mitigate clinically insignificant disease by defining the very mild disease category (Messidor 1) as normal, there was still the possibility of clinically very mild disease in the most severe Messidor category (eg, 16 microaneurysms is Messidor 3 but could be considered minimal retinopathy on most clinical grading scales). Since our sensitivity analysis suggested slightly better diagnostic accuracy when completely excluding all Messidor level 1 images, we believe our dichotomization was appropriately conservative.

There are several potential benefits to the use of crowdsourcing for the interpretation of visual data in ophthalmology. First, an inexpensive, rapid, and accurate system to reduce the number of images needing human grading in large public health screenings is needed. Importantly, this model should also be scalable; although the cost of grading per image here was greater than we have previously reported due to increased Amazon fees, the Turker compensation may have some elasticity which could be formally tested in the future. Despite these increased costs, crowdsourcing may be less expensive than other models of automated retinal image analysis and may be combined with other models to save costs and increase scalability. An approach which accurately identifies normal (or very mildly abnormal, allowing for some false negatives) fundi would be of great value and could reduce the skilled grader burden by up to 26% to 38% or more according to some investigators using artificial intelligence programs [19]. A first pass to remove normal images is currently being done with an artificial intelligence solution in Scotland’s national screening program [46]. A similar first pass or low-level annotation scheme was also validated using crowdsourcing to improve the accuracy and efficiency of expert grading of pathology slides for breast cancer [22]. If appropriately validated, crowdsourcing retinal images could provide a similar service at lower cost and with less infrastructure in all settings but could be particularly attractive in resource-poor settings. Likewise, a means to rapidly interrogate existing datasets could allow for nimble hypothesis generation for secondary data analyses. Overall, our results suggest that generating weighted classifications with Rasch analysis, which are then used in a weighted logistic regression model, may improve the accuracy of information obtained by crowdsourcing to grade retinal images for diabetic retinopathy.

## Appendix

Multimedia Appendix 1 Screenshot of the Amazon Mechanical Turk Web interface for fundus photo grading.

Multimedia Appendix 2 Screenshot of the Volunteer Science hosted Web interface for fundus photo grading.

## Abbreviations

AMT: Amazon Mechanical Turk
AUROC: area under the receiver operating characteristic
DM: diabetes mellitus
DR: diabetic retinopathy
HIT: human intelligence task
ICTR: Institute for Clinical and Translational Research
IRB: institutional review board
MV: majority vote
NCATS: National Center for Advancing Translational Sciences
ROC: receiver operating characteristic
