# Supplementary figures and images for: Improving Consensus Scoring of Crowdsourced Data Using the Rasch Model: Development and Refinement of a Diagnostic Instrument
Source: J Med Internet Res. 2017 Jun 20;19(6):e222. doi: 10.2196/jmir.7984 (PMC5497070; doi:10.2196/jmir.7984)

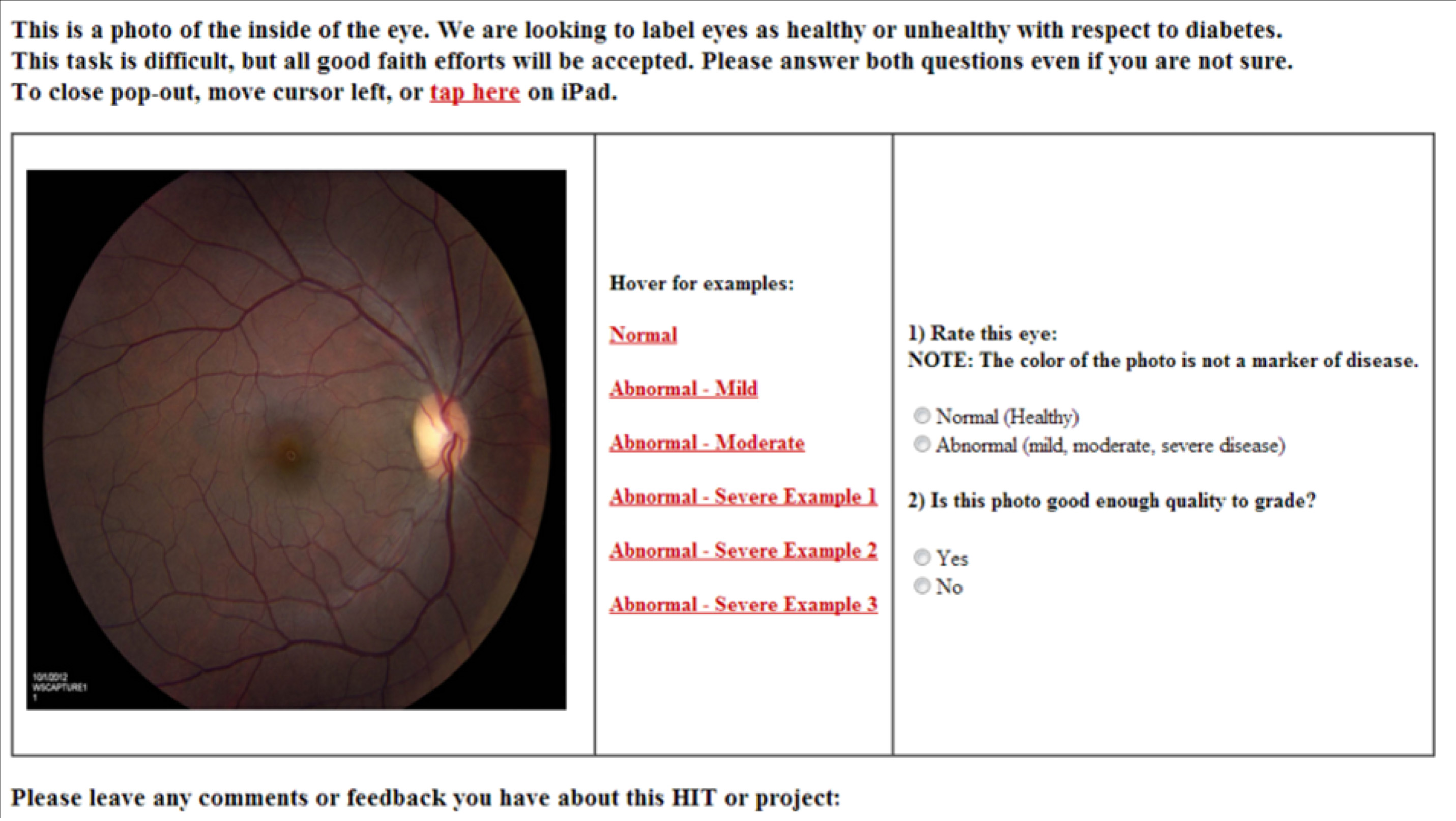

Supplement: Multimedia Appendix 1 [file jmir_v19i6e222_app1.jpg]

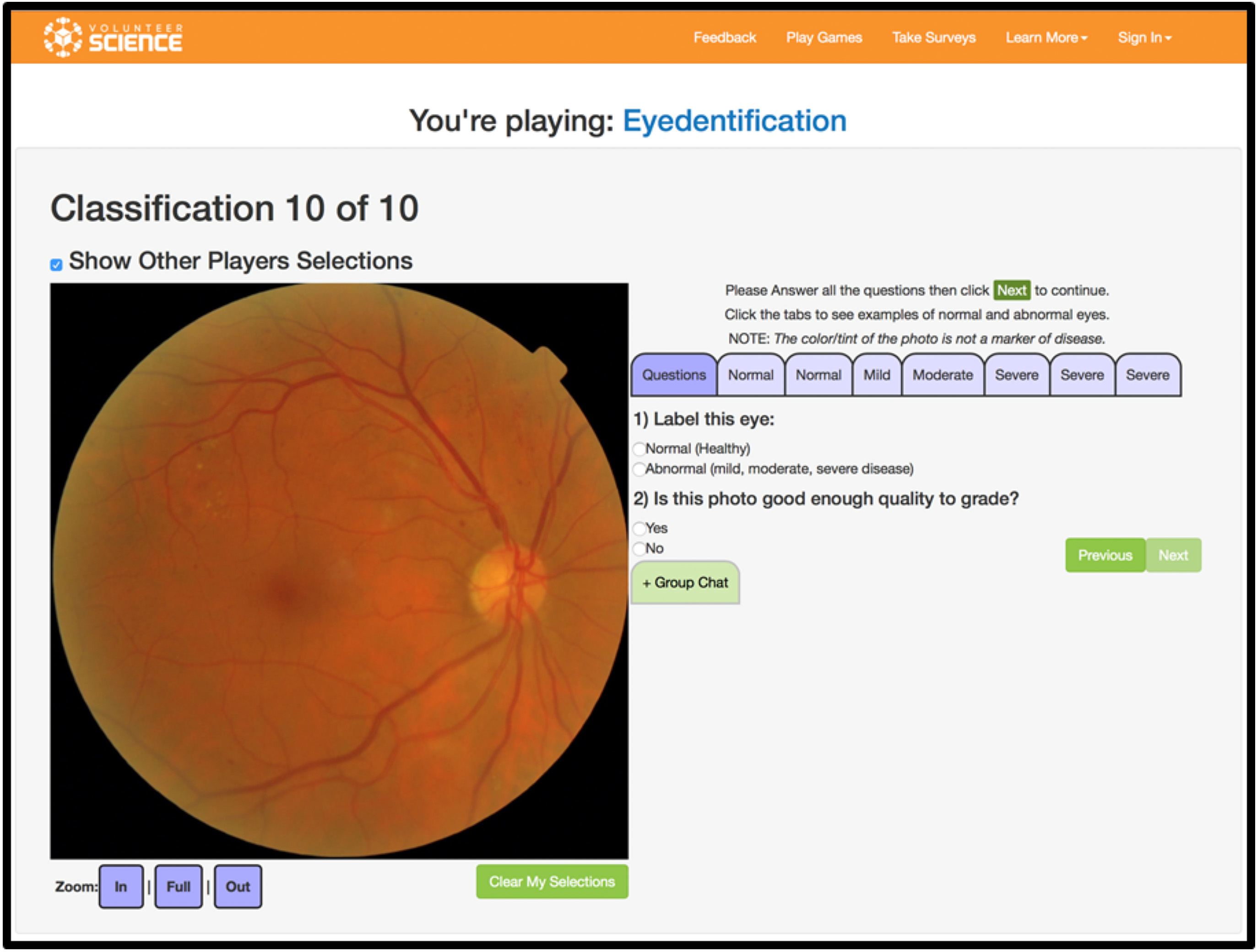

Supplement: Multimedia Appendix 2 [file jmir_v19i6e222_app2.jpg]
